# Ribosomal protein S27-like regulates autophagy via the β-TrCP-DEPTOR-mTORC1 axis

**DOI:** 10.1038/s41419-018-1168-7

**Published:** 2018-11-13

**Authors:** Xiufang Xiong, Xia Liu, Haomin Li, Hengqian He, Yi Sun, Yongchao Zhao

**Affiliations:** 1 Institute of Translational Medicine, Zhejiang University School of Medicine, Hangzhou, 310029 China; 2 Key Laboratory of Combined Multi-Organ Transplantation, Ministry of Public Health, First Affiliated Hospital, Zhejiang University School of Medicine, Hangzhou, China; 3 Division of Radiation and Cancer Biology, Department of Radiation Oncology, University of Michigan, Ann Arbor, MI USA; 4 Children’s Hospital, Zhejiang University School of Medicine, Hangzhou, Zhejiang China

## Abstract

RPS27L (Ribosomal protein S27-like), an evolutionarily conserved ribosomal protein, is a p53 target and a physiological p53 regulator. We previously reported that *Rps27l* disruption enhanced lymphomagenesis in *Trp53*^*+/*^^−^ mice by triggering genome instability and sensitized *Trp53*^*+/*^^−^ mice to radiation by blocking DNA damage response. Whether and how RPS27L modulates autophagy is totally unknown. Here we report that RPS27L silencing significantly induced autophagy in breast cancer MB231 and SK-BR3 cells harboring mutant p53. Mechanistically, RPS27L silencing remarkably inactivated mTORC1, a major negative autophagy regulator, but not mTORC2. Autophagy induction and mTORC1 inactivation was also observed in MEFs with *Rps27l* deletion. More specifically, RPS27L silencing shortened the protein half-life of β-TrCP, a substrate receptor of Skp1-Cullin 1-F-box (SCF) ubiquitin ligase, which is responsible for DEPTOR degradation, leading to DEPTOR accumulation to inhibit mTORC1 activity. Furthermore, RPS27L silencing-induced autophagy and mTORC1 inactivation can be partially rescued by simultaneous DEPTOR silencing, suggesting a causal role of DEPTOR. Biologically, autophagy inhibitor, chloroquine (CQ), or Bafilomycin A1 (BAF A1), significantly induced apoptosis in RPS27L silenced cells, indicating that autophagy is a cellular survival mechanism in response to RPS27L loss. Finally, RPS27L levels were reduced in human breast cancers, as compared to adjacent normal tissues. Collectively, our study suggests that RPS27L reduction might play a promoting role during breast tumorigenesis by autophagy induction via the β-TrCP-DEPTOR-mTORC1 axis.

## Introduction

Ribosomal proteins, a family of RNA-binding proteins, play the essential roles in the ribosomal biogenesis, a tightly regulated process to assemble ribosomes for protein synthesis required for the life cycle of a cell^1,2^. In addition, increasing evidence revealed that ribosomal-free ribosomal proteins, which accumulate by disruption of ribosomal biogenesis in response to numerous extracellular or intracellular stimuli, have multiple extraribosomal functions, including regulation of apoptosis, cell cycle progression, cell proliferation, genomic stability, neoplastic transformation, immune signaling, development, among others (for review, ref. ^3,4^). Thus, the dysregulation of ribosomal protein contributes to abnormal cell growth, eventually leading to tumorigenesis.

It has been documented that ribosomal proteins play important roles in the genesis and progression of breast cancer, one of the most common cancers diagnosed in women around the world^5^. For example, knockdown of RPS3^6^, RPS6^7^, and RPL24^8^ in breast cancer cells inhibited cell growth, viability, or proliferation, or induced apoptosis in cell based studies. Moreover, downregulation of RPS19 or RPL39 suppressed breast tumor growth and progression in a transgenic breast cancer model, or tumor initiation and lung metastasis in patient-derived and human breast cancer xenografts, respectively^9,10^. In contrast, silencing of RPL5 in breast cancer cells promoted cell cycle progression and accelerated tumor progression in a xenograft mouse model^11^. Overexpression of RPL19 activated the unfolded protein response and sensitized MCF7 breast cancer cells to endoplasmic reticulum stress-induced cell death^12^. Thus, individual ribosomal protein displays distinct function, playing either tumor suppressive or tumor promoting roles in breast cancer.

RPS27L (Ribosomal protein S27-like, NM_015920), an evolutionarily conserved ribosomal protein in 40 S small subunit, differs from its family member RPS27 (NM_001030) only by three amino acids (R5K, L12P, K17R) at its N-terminus. We and the others have previously reported that RPS27L is a direct p53 transcriptional target^13,14^. Our knockout mouse study demonstrated that Rps27l plays a critical role in postnatal development by modulating p53 levels. Specifically, Rps27l disruption triggers ribosomal stress to activate p53 by stabilizing Mdm2, which degrades Mdm4 to reduce Mdm2-Mdm4 E3 ligase activity towards p53, leading to p53-dependent postnatal death via apoptosis, which can be rescued by heterozygous deletion of *Trp53*^15^. Interestingly, *Rps27l* deletion causes genomic instability to selectively lose *Trp53* heterozygosity, resulting in spontaneous lymphomagenesis in *Trp53*^*+/*^^−^ background, suggesting the tumor suppressive role of Rps27l in vivo^15^. Furthermore, disruption of Rps27l impairs DNA damage response to sensitize *Trp53*^*+/*^^−^ mice to ionizing radiation^16^. However, whether and how RPS27L acts as a tumor suppressive or tumor promoting factor in breast cancer is previously unknown.

In this study, we report RPS27L silencing significantly induced autophagy in breast cancer cells as well as mouse fibroblasts by selectively inactivating mTORC1. Mechanistically, RPS27L silencing caused the accumulation of DEPTOR, a direct mTOR negative regulator^17^, by reducing the levels and half-life of β-TrCP, which is responsible for DEPTOR degradation^18–20^. Blockage of autophagy induced by RPS27L silencing significantly enhanced apoptosis. Finally, RPS27L level was significantly reduced in human breast cancer tissues. Collectively, it appears that RPS27L downregulation during breast tumorigenesis could serve as a survival signal to facilitate tumor formation. Thus, RPS27L might play a tumor suppressive role in breast tumorigenesis by inhibiting autophagy.

## Results

### RPS27L silencing induces autophagy in breast cancer cells

**Fig. 1:**
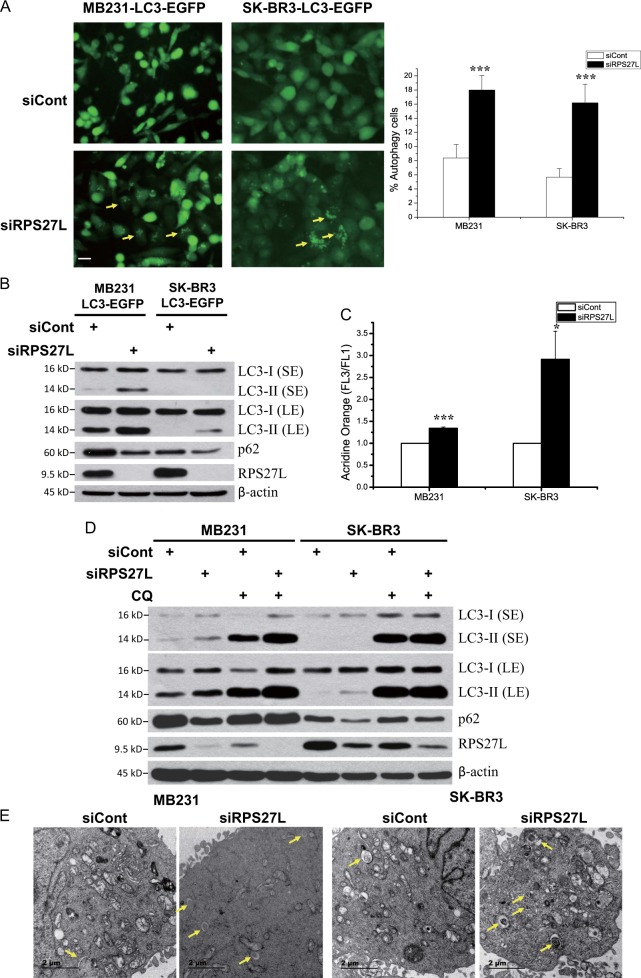
Silencing of RPS27L induces autophagy in breast cancer cells. **a** Autophagy measured by appearance of punctate vesicle structure. MB231 and SK-BR3 cells stably expressing EGFP-LC3 were transfected with scramble control siRNA (siCont) or siRNA targeting RPS27L for 48 h before photography under a fluorescent microscope (left panel). Size bar = 20 μm. Cells with punctate vesicle structures of EGFP-LC3 were counted and expressed as percentage of autophagy cells (right panel). ***p < 0.001. **b** Autophagy measured by LC3-II conversion and p62 degradation. Cells were transfected with scramble control siRNA or siRNA targeting RPS27L for 48 h, followed by immunoblotting (IB) with indicated antibodies (Abs). *SE* shorter exposure, *LE* longer exposure. **c** Autophagy measured by acridine orange staining. Cells were transfected with indicated siRNA oligos for 48 h, followed by acridine orange staining and flow cytometry, using the ratio between geo-mean fluorescence intensity of red vs. green fluorescence (FL3/FL1) to quantify autophagy. Shown are mean ± S.E.M. from three independent experiments. *p < 0.05, ***p < 0.001. **d** Autophagy induction by RPS27L silencing was blocked by CQ treatment. Cells were transfected with indicated siRNA oligos for 48 h, and then left untreated or treated with 50 μM CQ for 24 h, followed by IB with indicated Abs. **e** Autophagosomes detected by transmission electron microscopy (TEM). Cells were transfected with indicated siRNA oligos for 48 h, followed by TEM analysis. Autophagosomes were indicated by arrows. Size bar = 2 μm

In our effort to determine potential effect of RPS27L on the growth of two breast cancer cell lines, we found that siRNA-based RPS27L silencing significantly triggered autophagy, an evolutionarily conserved catabolic degradation process to clear damaged organelles and recycle nutrients for the maintenance of cellular homeostasis and the adaptation to various stresses^21,22^, which has been previously shown to play important roles in breast cancer^23,24^. Specifically, RPS27L silencing significantly induced autophagy punctate vesicle structure in MB231 and SK-BR3 cells stably expressing EGFP-LC3 (Fig. 1a). Biochemically, RPS27L silencing-induced LC3 conversion (LC3-I to LC3-II) and p62 degradation (Fig. 1b), two widely used markers of autophagy^25^. We further used another different siRNA oligos targeting RPS27L and confirmed the autophagic changes in LC3 and p62 (Figure S1A). Consistently, acridine orange staining analysis by flow cytometry also demonstrated that autophagic acidic vesicular organelles in both of parental cell lines were significantly increased upon silencing of RPS27L (Fig. 1c). Furthermore, we found that both LC3-II and p62 degradation were abrogated by chloroquine (CQ), a lysosomotropic agent, or bafilomycin A1 (BAF A1), a vacuolar H^+^-ATPase inhibitor to inhibit lysosomal degradation (Fig. 1d, Figure S1B), suggesting that RPS27L silencing acts at the initiation, but not degradation stage of autophagy. Finally, examination with transmission electron microscopy revealed more autophagosomes in both cell lines upon RPS27L silencing (Fig. 1e). Interestingly, silencing of RPS27, a family member of RPS27L, which differs from RPS27L only by three amino acids at its N-terminus, failed to induce autophagy, as evidenced by lack of LC3 conversion and p62 reduction (Figure S1C), suggesting a specific role of RPS27L in autophagy regulation. In addition, RPS27L silencing also reduced cell growth in both MB231 and SK-BR3 cells (Figure S1D). Collectively, these results demonstrated that RPS27L silencing readily induces autophagy in breast cancer cells.

### RPS27L silencing inhibits the activity of mTORC1, but not mTORC2

**Fig. 2:**
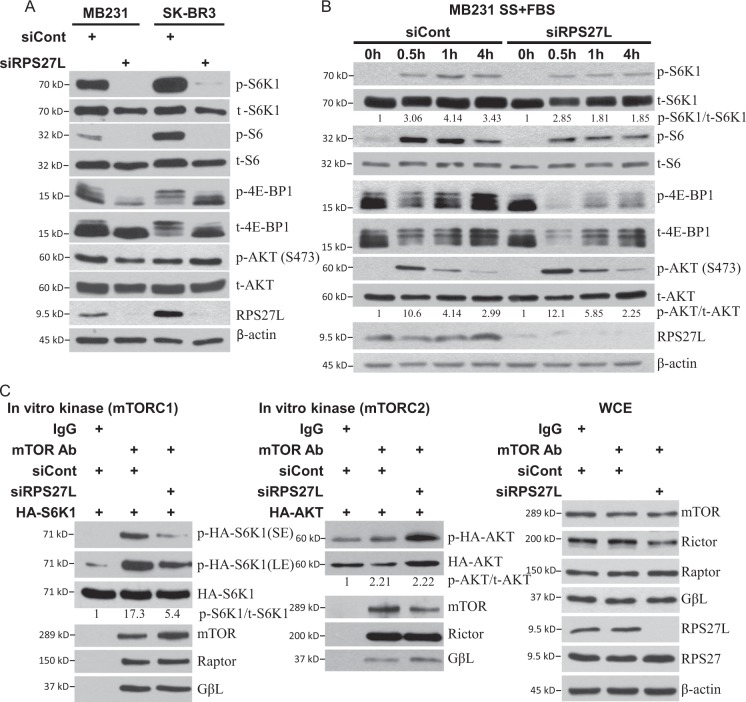
Silencing of RPS27L inhibits mTORC1 activity in breast cancer cells. **a**, **b** Silencing of RPS27L inhibits the phosphorylation of mTORC1 downstream effectors. Cells were transfected with scramble control siRNA or siRNA targeting RPS27L for 48 h, followed by IB with indicated Abs. **b** Silencing of RPS27L inhibits the phosphorylation of mTORC1 downstream effectors triggered by serum addition after serum starvation. Cells were transfected with indicated siRNA oligos for 48 h, and then serum starved for 24 h, followed by addition of serum for indicated time periods. Cells were then harvested for IB with indicated Abs. Densitometry quantification was performed with ImageJ. The ratios of phosphorylated levels and total protein levels were shown. *SS* serum starvation, *FBS* fetal bovine serum. **c** Silencing of RPS27L inhibits mTORC1 activity, but does not affect mTORC2 activity in in vitro kinase activity assays. HA-tagged S6K1 or HA-tagged AKT1 was transfected into 293 cells, followed by immune-affinity purification by immunoprecipitation using bead-conjugated HA antibody. After elution with HA peptide, HA-S6K1 (left panel), or HA-AKT1 (middle panel) was added into a kinase reaction mixture containing mTOR complex, which was immunoprecipitated by anti-mTOR Ab from MB231 cells upon silencing of RPS27L for 48 h, along with siRNA control. The reaction mixture was incubated for 90 min at 30 °C with constant shaking, followed by IB with indicated Abs. The whole-cell extracts (WCE) (right panel) of MB231 cells were subjected to IB with indicated Abs. Densitometry quantification was performed with ImageJ. The ratios of phosphorylated levels and total protein levels were shown

We next investigated whether autophagy induced by RPS27L silencing is mediated by inactivation of mTORC1, a master negative regulator of autophagy^26^. Indeed, RPS27L depletion significantly decreased phosphorylation of S6K1 (ribosomal protein S6 kinase 1) and 4E-BP1 (eukaryotic initiation factor 4E-binding protein 1), two well-characterized downstream effectors of mTORC1 and often used as a readout of mTORC1 activity^27^, without affecting phosphorylation of AKT^S473^, a well-characterized substrate of mTORC2^28^ (Fig. 2a). Consistently, the phosphorylation of S6, the downstream target of S6K1, was also decreased upon RPS27L depletion (Fig. 2a). We further examined if RPS27L depletion also blocked mTORC1, activated by serum or TPA, a mitogen. Following RPS27L silencing, cells were serum starved for 24 h, and then exposed to serum or TPA for various time points. RPS27L depletion inhibited activation of mTORC1, but had no effect on mTORC2, as reflected by reduced levels of phosphorylation of S6K1, S6, and 4E-BP1, but unchanged level of pAKT^S473^ (Fig. 2b, Figure S2). Finally, an in vitro kinase assay showed that S6K1 phosphorylation mediated by mTORC1 complex isolated from cells with RPS27L silencing was remarkably lower than that from the control siRNA, whereas no difference was seen for AKT phosphorylation (Fig. 2c). Thus, RPS27L silencing selectively suppresses the activity of mTORC1, but not mTORC2.

**Fig. 3:**
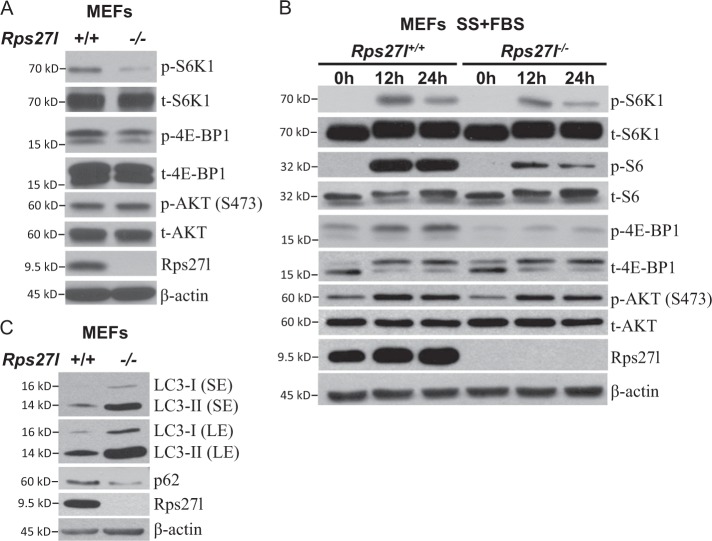
*Rps27l* disruption inhibits mTORC1 activity and induces autophagy in MEF cells. **a**, **b** Reduced phosphorylation of mTORC1 downstream effectors in *Rps27l*^−^^*/*^^−^ MEFs. *Rps27l*^*+/+*^ or *Rps27l*^−^^*/*^^−^ MEFs were left untreated (**a**) or serum starved for 24 h, followed by addition of serum for indicated time periods (**b**). Cells were then harvested for IB with indicated Abs. **c** Autophagy measured by LC3-II conversion and p62 degradation in MEFs. *Rps27l*^*+/+*^ or *Rps27l*^−^^*/*^^−^ MEFs were generated and harvested for IB with indicated Abs

We further extended the findings in breast cancer cells to normal MEF cells, by using two independent pairs of *Rps27l*^*+/+*^ vs. *Rps27l*^−^^*/*^^−^ MEFs^15^, and found that *Rps27l*^−^^*/*^^−^ MEFs had reduced basal phosphorylation levels of S6K1 and 4E-BP1 (Fig. 3a, Figure S3). Like in breast cancer cells, the activation of mTORC1 by serum was also inhibited in *Rps27l*^−^^*/*^^−^ MEFs, as reflected by the reduced phosphorylation of S6K1, S6, and 4E-BP1 upon addition of serum after serum starvation (Fig. 3b), whereas the phosphorylation of AKT showed no difference regardless of Rps27l status (Fig. 3b), indicating that *Rps27l* disruption indeed inactivates mTORC1, but has no effects on mTORC2. Furthermore, LC3 conversion and p62 degradation were readily detected in *Rps27l*^−^^*/*^^−^ MEFs (Fig. 3c). Taken together, our study strongly suggests that RPS27L regulation of autophagy is a general phenomenon.

### RPS27L silencing shortens protein half-life of β-TrCP, leading to DEPTOR accumulation to inactivate mTORC1 and induce autophagy

**Fig. 4:**
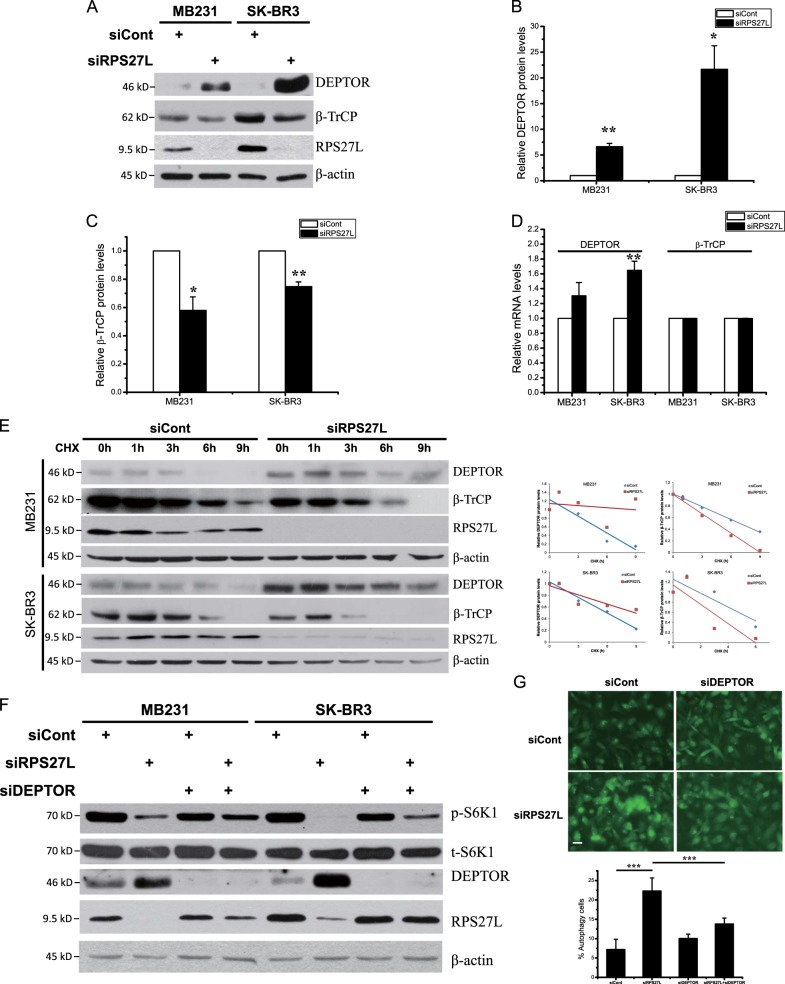
Silencing of RPS27L triggers β-TrCP degradation, leading to DEPTOR accumulation. **a**–**c** Silencing of RPS27L increases DEPTOR levels, but decreases β-TrCP levels. Cells were transfected with scramble control siRNA or siRNA targeting RPS27L for 48 h, followed by IB with indicated Abs (**a**). Densitometry quantification of DEPTOR (**b**) and β-TrCP (**c**) was performed with ImageJ (*n* = 3). Shown are mean ± S.E.M. *p < 0.05, **p < 0.01, ***p < 0.001. **d** Silencing of RPS27L slightly increases DEPTOR mRNA levels, but has no effects on β-TrCP mRNA levels. Cells were transfected with indicated siRNA oligos for 48 h, followed by qRT-PCR (*n* = 3). Shown are mean ± S.E.M. **p < 0.01. **e** Silencing of RPS27L extends DEPTOR protein half-life, but shortens β-TrCP protein half-life. Densitometry quantification was performed with ImageJ, and the decay curves are shown (right). **f** Silencing of DEPTOR reverts the reduction of S6K1 phosphorylation upon RPS27L silencing. Cells were transfected with scramble control siRNA or indicated siRNA for 48 h, followed by IB with indicated Abs. **g** Silencing of DEPTOR reverts autophagic induction upon RPS27L silencing. MB231 cells stably expressing EGFP-LC3 were transfected with scramble control siRNA or indicated siRNA for 48 h before photography under a fluorescent microscope (top panel). Size bar = 20 μm. Cells with punctate vesicle structures of EGFP-LC3 were counted and expressed as percentage of autophagy cells (bottom panel). ***p < 0.001

In the process of in vitro kinase assays, we noticed that RPS27L depletion did not affect the levels of mTOR and its components, nor mTOR interaction with its components in mTOR complexes, including RAPTOR, RICTOR, and GβL (Fig. 2c). To elucidate the mechanism of mTORC1 inactivation, we focused on DEPTOR, a naturally occurring inhibitor of mTOR^17^. By western blotting, we found that in both MB231 and SK-BR3 cells RPS27L silencing significantly upregulated the protein levels of DEPTOR (Fig. 4a, b), but only slightly increased DEPTOR mRNA levels by qRT-PCR analysis (Fig. 4d). The half-life of DEPTOR was significantly extended upon RPS27L silencing, suggesting RPS27L knockdown inhibited DEPTOR degradation, leading to its accumulation (Fig. 4e). We then examined potential change of β-TrCP, the F-box protein which binds to DEPTOR for its targeted degradation by SCF E3 ubiquitin ligase^18–20^, and found RPS27L silencing significantly decreased the levels of β-TrCP (Fig. 4a, c) by shortening its protein half-life (Fig. 4e), but has no effects on its mRNA levels (Fig. 4d). We next determined contribution of accumulated DEPTOR in mTORC1 inactivation and autophagy induction by RPS27L knockdown, and found that RPS27L-triggered reduction in S6K1 phosphorylation (Fig. 4f), and induction in autophagy punctate vesicle structure in MB231 cells stably expressing EGFP-LC3 (Fig. 4g) can be partially rescued by simultaneous silencing of DEPTOR, indicating a causal role. Thus, RPS27L silencing decreases β-TrCP levels by shortening its protein half-life, leading to DEPTOR accumulation to inactivate mTORC1 and induce autophagy.

### Blockage of autophagy induced by RPS27L silencing inhibits cell growth by triggering apoptosis

**Fig. 5:**
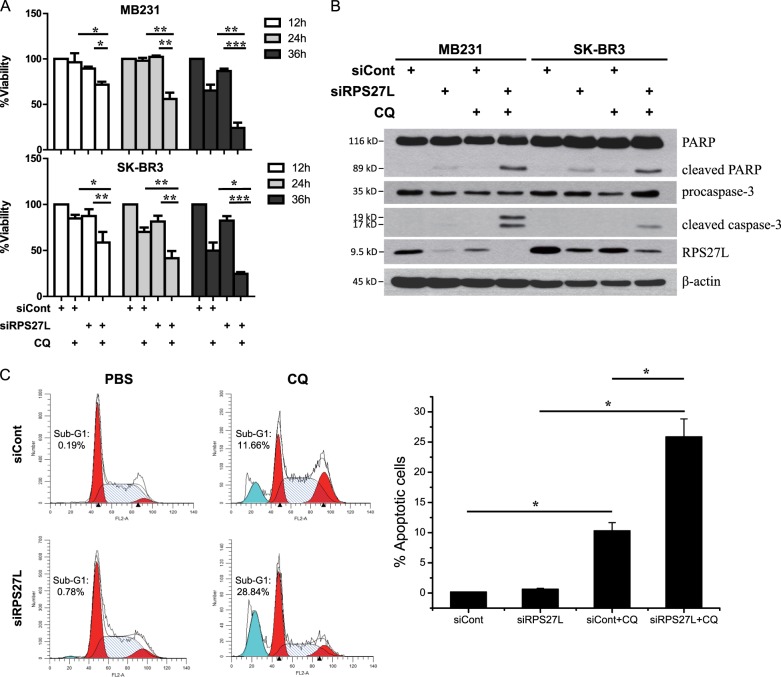
Blockage of autophagy by CQ treatment significantly induces cell apoptosis upon silencing of RPS27L. **a** CQ-siRPS27L combination significantly suppressed growth of MB231 and SK-BR3 cells. Cells were transfected indicated siRNA oligos for 48 h, then left untreated or treated with CQ treatment for indicated time periods, followed by ATPlite assay. Data shown are mean ± SEM. *n* = 3, *p < 0.05, **p < 0.01, ***p < 0.001. **b**, **c** CQ-siRPS27L combinational treatment caused a higher induction of cell apoptosis. MB231 (**b**) and SK-BR3 cells (**b**, **c**) were transfected indicated siRNA oligos for 48 h, and then left untreated or treated with 50 µM CQ for 24 h, followed by IB with indicated Abs (**b**), or by flow cytometry (**c**) (left, a representative FACS profile, and right, the percentage of cells at sub-G1 phase, mean ± S.E.M.; *n* = 3; *p < 0.05.)

We next determined whether induction of autophagy upon RPS27L silencing affects cell growth by blocking autophagy via small molecule inhibitors, and found that while CQ alone treatment had minimal effect on cell growth, combination of CQ with siRPS27L significantly suppressed growth of MB231 and SK-BR3 cells (Fig. 5a). To determine the nature of growth suppression, we performed western blotting to determine the cleavage of PARP and caspase-3 as the readout for apoptosis, and found that while RPS27L depletion or CQ treatment caused minor or no cleavage, their combination remarkably enhanced cleavage (Fig. 5b). Furthermore, we measured the percentage of cells at sub-G1 phase by flow cytometry as another independent marker for apoptosis, and found that the combinational treatment caused a much higher induction of sub-G1 population (Fig. 5c). Finally, we confirmed that blockage of RPS27L silencing-induced autophagy by BAF A1 also enhanced apoptosis, as evidenced by the cleavage of PARP and caspase-3 (Figure S4). Taken together, abrogation of autophagy induced by RPS27L depletion enhances cell killing via induction of apoptosis, suggesting that autophagy triggered by RPS27L silencing is a cellular survival response of breast cancer cells.

### RPS27L level is reduced in human breast cancer tissues

**Fig. 6:**
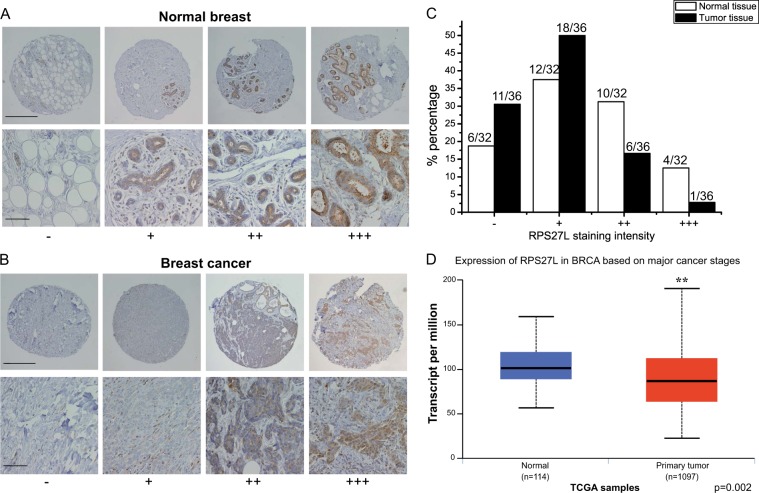
RPS27L levels were reduced in human breast cancer tissues. **a**, **b** RPS27L staining in normal breast tissues (**a**) and breast cancer tissues (**b**). Breast tissue microarrays containing normal breast and tumor tissues were stained for RPS27L expression. Stained normal and tumor tissues were classified into four groups (− to +++) according to the staining intensity. Scale bar in top panels = 500 μm, scale bar in bottom panels = 100 μm. **c** The percentage of normal or tumor tissues in each staining group. Tissue samples with different staining intensity were grouped and indicated. **d** RPS27L transcript was significantly reduced in primary breast tumors from TCGA database. *p* = 0.002

Our previous studies showed that *Rps27l* is required for genomic stability under a *Trp53*^*+/*^^−^ background, and *Rps27l*^−^^*/*^^−^*;Trp53*^*+/*^^−^ mice spontaneously develop lymphoma^15^ and are extremely sensitive to ionizing radiation^16^, suggesting RPS27L as a putative tumor suppressor in vivo. We therefore determined potential alterations of RPS27L levels in breast cancer tissues, as compared to adjacent normal tissues. We immuno-stained human normal breast tissue and breast cancer tissue microarrays, consisting of 32 normal breast tissues and 36 tumor tissues. Based on the staining intensity, we classified the tissues into four groups, with group 1 showing no or minimal staining (−) and group 4 the highest staining (+++; Fig. 6a, b). We found that RPS27L levels in normal breast tissues were mainly in group 2 (12 of 32, 37%) and group 3 (10 of 32, 31.2%), with 4 samples in group 4 (12.5%), whereas most breast tumor tissues were classified into group 1 (11 of 36, 30.6%) and group 2 (18 of 36, 50%), and only one tumor tissue showed highest staining intensity (Fig. 6c). Thus, RPS27L staining intensity was lower in breast tumors, as compared to normal breast tissues. Consistently, using TCGA transcriptome data, the transcripts per million (TPM) expression value was obtained from estimation of transcripts generated from the gene multiplied by 10^6^ ^29^. The statistical analysis by *t*-test between TPM data of breast carcinoma tissues (*n* = 1097) and normal tissues (*n* = 114) showed that the levels of RPS27L expression were significantly downregulated in breast carcinoma (Fig. 6d). Collectively, the expression of RPS27L is decreased in breast tumors, suggesting that the reduction of RPS27L expression could play a role in breast tumorigenesis.

## Discussion

In this study, we made a novel observation that RPS27L depletion via siRNA-based silencing in breast cancer cells significantly induced autophagy by following lines of evidence: (1) the appearance of autophagy punctate vesicle structure visualized by EGFP-LC3, (2) the increase of autophagic acidic vesicular organelles stained by acridine orange, (3) increased number of autophagosomes detected by transmission electron microscopy, (4) the conversion of LC3-II from LC3-I, and (5) p62 degradation, measured by western blotting (Fig. 1). Moreover, *Rps27l* deletion also induces autophagy in normal mouse embryonic fibroblasts, as evidenced by LC3 conversion and p62 degradation (Fig. 3). These results suggest that RPS27L is a general regulator of autophagy. In consistent with our finding, induction of autophagy was also observed upon manipulation of several other ribosomal proteins. For example, depletion of RPLP proteins, including RPLP0, RPLP1, and RPLP2, induced autophagy in breast and ovarian cancer cell lines^30^. Autophagy was also seen in lymphoblastoid cell lines derived from patients with Diamond–Blackfan anemia carrying mutations causing haploinsufficiency of *RPS17*, *RPL11*, or *RPS7*; in CD34^+^ erythrocyte progenitor cells with silencing of RPS19; and in red blood cells of zebrafish embryos with haploinsufficient *RPS7*^31^. Thus, induction of autophagy may be a general cellular response to various stresses induced by mutations or deficiency of ribosomal proteins in multiple cell types under physiological or pathological conditions.

It is well established that autophagy is negatively regulated by mTOR signaling pathway, in which mTORC1 triggers the phosphorylation of multiple autophagy-related proteins, such as ULK1/2 and ATG13, to inhibit autophagy initiation and autophagosome nucleation, whereas mTORC2 indirectly suppresses autophagy by phosphorylating AKT at S473, which ultimately activates mTORC1^26^. We found that RPS27L silencing remarkably inhibited the activity of mTORC1, but not mTORC2, in breast cancer cells and MEFs, as evidenced by reduced phosphorylation levels of their downstream effectors as well as direct in vitro kinase activity assays (Figs. 2 and 3). DEPTOR, a naturally occurring inhibitor of mTOR via directly binding to both mTORC1 and mTORC2^17^, was accumulated upon silencing of RPS27L (Fig. 4). It was previously reported that expression of DEPTOR was negatively regulated by mTOR at the transcriptional levels and by β-TrCP at post-translational levels^17–20^. Indeed, we detected DEPTOR increase slightly at the mRNA levels and significantly at the protein levels when RPS27L was silenced to inactivate mTORC1 (Fig. 4), suggesting that the induction of DEPTOR mainly occurred at post-translational levels. We then focused on β-TrCP, which binds to DEPTOR and promotes its degradation^18–20^. Indeed, we found that RPS27L depletion reduced protein levels of β-TrCP by shortening its protein half-life, without affecting its mRNA levels (Fig. 4). The stability of β-TrCP was reported to be subjected to the regulation by several E3 ubiquitin ligases, including SMURF2^32^, SKP2^33^, and SAG-CRL5^34^. At the present time, it is unknown how siRPS27L promotes β-TrCP degradation. It is possible, however, that RPS27L depletion blocks mTORC1/S6K signaling and serves as a feedback loop to control β-TrCP1 stability (Figs. 2a and 4a), a subject for future investigation.

Autophagy is an evolutionarily conserved catabolic degradation process, in which cytoplasmic cargo is engulfed by double-membrane autophagosomes for subsequent degradation in autolysosomes fused by lysosomes with autophagosomes, followed by release to recycle components^21,22^. Autophagy plays paradoxical roles in breast cancer initiation and progression^24^. A tumor suppressive role of autophagy in breast cancer was demonstrated by the development of spontaneous mammary hyperplasia in mice with heterozygous deletion of Beclin 1, an autophagy gene which is mono-allelically deleted in human breast^35^. However, accumulating data show that autophagy promotes breast tumorigenesis by facilitating the adaptation and survival of cancer cells in response to metabolic and genotoxic stress^23^. In our study, blockage of autophagy induced by RPS27L silencing significantly inhibits the growth of breast cancer cell via the induction of apoptosis (Fig. 5), indicating that autophagy plays a survival role in response to stress triggered by RPS27L deficiency. Thus, our study suggest abrogation of autophagy by autophagy inhibitors, which is considered as an attractive approach for breast cancer therapy^23,24^, may have therapeutic value in the treatment of breast cancer patients with low RPS27L expression.

Altered expression or gene mutations in several ribosomal proteins were reported in breast cancers. For instance, RPS19^9^, RPL19^12^, RPL24^8^, and RPLP^30^ were found to be upregulated, whereas RPL5^11^, RPL22^36^, and RPL41^37^ were downregulated in human breast cancer tissues, as compared to normal tissues. Moreover, RPL39 mutation^10^, low RPL5 expression^11^, or downregulation of RPS16^38^, was reported to be negatively associated with the survival of breast cancer patients. In the present study, by immunohistochemistry analysis of breast tissue microarray and statistical analysis of TCGA transcriptome data, we showed that the expression of RPS27L was also lower in breast tumors than in normal breast tissues (Fig. 6), suggesting it may play a role in breast tumorigenesis. The correlation of RPS27L expression with human cancer was also reported in colorectal cancer, in which low expression of RPS27L in either feces or cancer tissues was related to a worse patient prognosis^39^. Given that *Rps27l* deletion accelerates the development of spontaneous lymphoma in *Trp53*^*+/*^^−^ mice^15^, and that RPS27L downregulation triggers protective autophagy (this study), it is likely that RPS27L play a general role as a tumor suppressor, and its inactivation facilitates tumor development.

In summary, we made here a novel observation that RPS27L depletion triggers protective autophagy by mTORC1 inactivation via DEPTOR accumulation as a result of β-TrCP reduction. Our study established a previously unrealized active axis of RPS27L-β-TrCP-DEPTOR-mTORC1 in regulation of autophagy. Given the fact that blockage of autophagy triggers apoptosis in breast cancer cells with RPS27L depletion, and breast cancer tissues have in general lower RPS27L expression, our study may provide a rationale for enhancing the efficacy of autophagy inhibition in anticancer therapy for human breast cancers with RPS27L downregulation.

## Materials and methods

### Cell culture

SK-BR3, MB231 human breast cancer cells and 293 human embryonic kidney cells were maintained in Dulbecco’s modified Eagle’s medium (DMEM) supplemented with 10% (v/v) fetal bovine serum (FBS). SK-BR3 and MB231 cells stably expressing EGFP-LC3 were established and maintained as described^40^. *Rps27l*^*+/+*^ or *Rps27l*^−^^*/*^^−^ MEFs cells as described^15^ were cultured in DMEM with 15% FBS, 2 mM l-Glutamine, 0.1 mM MEM non-essential amino acids at 37 °C in a 5% CO_2_ humidified chamber.

### siRNA silencing

Cells were transfected with the following siRNA oligonucleotides by Lipofectamine 3000 (Invitrogen, CA, USA). siRPS27L-1: 5′-AAT GAT TCA AAC AGC TTC CTG-3′; siRPS27L-2: 5′-GTT GTC TCA CAG AAA GCC TTA-3′; siRPS27: 5′-AAG CAC TCT GAG TCA AGA TGA-3′; siDEPTOR: 5′-GCC ATG ACA ATC GGA AAT CTA-3′; and siCont: 5′-TTC TCC GAA CGT GTC ACG TTT-3′.

### Acridine orange immunofluorescent staining

Quantification of autophagy by acridine orange (AO) staining using flow cytometry was performed as described^40^. Briefly, cells were stained with 1 μM AO for 15 min at 37 °C, followed by flow cytometry. Autophagy was quantified as a ratio between geo-mean fluorescence intensity of red (650 nm) vs. green fluorescence (510–530 nm) (FL3/FL1), and the data are presented as the fold changes with an arbitrary setting of autophagy in cells transfected with scramble control siRNA as 1.

### Western blotting

Cells or tissues were harvested, lysed in a RIPA buffer with protease inhibitors and phosphatase inhibitors, and then subjected to western blotting as described^15^, using various antibodies as follows: RPS27L polyclonal rabbit antibody was raised and purified as described^13^, Phospho-4E-BP1, 4E-BP1, Phospho-S6K1 (Thr389), Phospho-AKT (Ser473), AKT, Phospho-S6, S6, β-TrCP, DEPTOR, mTOR, RICTOR, RAPTOR, GβL, PARP, cleaved caspase-3 (Cell Signaling Technology, MA, USA), β-actin, HA, LC3-I/II (Sigma, MO, USA), S6K1, mTOR (Santa Cruz Biotechnology, CA, USA), and p62 (MBL Life science, Japan).

### Transmission electron microscopy

Transmission electron microscopy (TEM) was performed as described previously^41^. Briefly, cells were fixed in 2.5% glutaraldehyde in PBS overnight, and then post-fixed in 1% OsO4 for 1 h followed by 2% uranyl acetate. After ethanol and acetone dehydration and embedding in polybed 812 resin (Sigma), thin sections (70 nm) were post-stained with 2% uranyl acetate followed by 0.3% lead citrate. The photos of sample sections were taken using a TECNAI 10 transmission electron microscope (FEI Company, Hillsboro, OR) at 120 kV.

### In vitro kinase assay

The in vitro kinase assay was performed as described^40^. Briefly, HA-tagged S6K1 or AKT1 was transfected into 293 cells for 48 h, followed by treatment with 20 μM LY294002 for 1 h before cell harvesting and lysis. HA-tagged S6K1 or AKT1 was pulled down by HA beads (Sigma, MO, USA) and eluted with HA peptide (APExBIO, TX, USA). The mTOR complex was purified from MB231 cells by immunoprecipitation using anti-mTOR antibody. HA-S6K1 or HA-AKT1 was incubated with bead-conjugated mTOR complex, respectively, in a kinase reaction buffer [25 mM HEPES (pH 7.4), 50 mM KCl, 10 mM MgCl_2_, 250 μM ATP] at 30 °C for 90 min with constant shaking. Phosphorylation of HA-S6K1 by mTORC1 or phosphorylation of HA-AKT1 by mTORC2 was detected by western blotting with phospho-S6K1 (T389) or phospho-AKT (S473) antibody, respectively.

### Quantitative RT-PCR

Total RNA was isolated from cells using a Trizol reagent (Invitrogen, CA, USA). Complementary DNA was made from RNA with PrimeScript™ RT reagent Kit (Perfect Real Time) (Takara Biotechnology, Dalian, China), and subjected to quantitative RT-PCR analysis, according to the manufacturer’s instruction of SYBR^®^ Premix Ex Taq^TM^ (Tli RNaseH Plus) (Takara Biotechnology, Dalian, China). The cycling program was set as follows: 37 °C for 15 min at RT, 95 °C for 30 s for the initial template denaturation and 40 cycles of denaturation at 95 °C for 5 s, annealing and extension at 60 °C for 30 s. The sequences of β-TrCP, DEPTOR, and GAPDH are as follows: β-TrCP-F: 5′-CCT CAT ACT TGC CCA GGA CC-3′, β-TrCP-R: 5′-AGG TGC AGA GGT GAA AGG AGG-3′; DEPTOR-F: 5′-GCA GCA GGA ATG AAG GTC TG-3′, DEPTOR-R: 5′-GTA TGT GCG GAG AAG ACT CGT AT-3′; GAPDH-F: 5′-GTT GCC ATC AAT GAC CCC TT-3′, GAPDH -R: 5′-GTG ATG GGA TTT CCA TTG AT-3′.

### ATPlite cell proliferation assay

A total of 5 × 10^3^ cells transfected with siRNA oligos were seeded in 96-well plates and treated with CQ for various time periods, followed by ATPlite assay for cell viability, according to the manufacturer’s instruction of ATPlite 1step Luminescence Assay System (PerkinElmer, MA, USA)^40^. The results from three independent experiments, each run in triplicate were plotted.

### Flow cytometry

Cells were treated with 50 µM CQ for 24 h, and then harvested and fixed in ice-cold 70% ethanol for overnight. Cells were stained with PI staining buffer, followed by flow cytometry.

### Human breast tissue microarray and immunohistochemistry

Human breast tissue microarrays were provided and stained with anti-RPS27L antibody^13^ by the University of Michigan Comprehensive Cancer Tissue Core. Briefly, after deparaffinization, rehydration, antigen retrieval and blocking, the arrays were incubated with RPS27L antibody at room temperature for 30 min on the DAKO AutoStainer using the DakoCytomation EnVision + System-HRP (DAB) detection kit, followed by counterstaining with hematoxylin. The stained slides were observed under a microscope (Olympus 1 × 71) and images were acquired using software DP controller (ver. 3.1.1.267, Olympus). Stained tissues were classified into four groups according to the staining intensity of each tissue.

### Statistical analysis

The two-tailed Student’s *t*-test for statistical analyses was performed using Prism 5 (GraphPad) for the comparison of parameters between groups. Statistical significance was determined as *p* < 0.05.

## Electronic supplementary material

Supplemental material

## References

[1] Lafontaine DLJ, Tollervey D (2001). The function and synthesis of ribosomes. Nat. Rev. Mol. Cell Biol..

[2] Fatica A, Tollervey D (2002). Making ribosomes. Curr. Opin. Cell Biol..

[3] Xu X, Xiong X, Sun Y (2016). The role of ribosomal proteins in the regulation of cell proliferation, tumorigenesis, and genomic integrity. Sci. China Life Sci..

[4] Zhou X, Liao WJ, Liao JM, Liao P, Lu H (2015). Ribosomal proteins: functions beyond the ribosome. J. Mol. Cell Biol..

[5] Amy MB, Rachel VS, Jessica ME, Adedayo AO (2014). BreastCancer biomarkers: risk assessment, diagnosis, prognosis, prediction of treatment efficacy and toxicity, and recurrence. Curr. Pharm. Des..

[6] Ono H (2017). Ribosomal protein S3 regulates XIAP expression independently of the NF-kappaB pathway in breast cancer cells. Oncol. Rep..

[7] Yi YW (2015). Dual inhibition of EGFR and MET induces synthetic lethality in triple-negative breast cancer cells through downregulation of ribosomal protein S6. Int. J. Oncol..

[8] Wilson-Edell KA (2014). RPL24: a potential therapeutic target whose depletion or acetylation inhibits polysome assembly and cancer cell growth. Oncotarget.

[9] Markiewski MM (2017). The ribosomal protein S19 suppresses antitumor immune responses via the complement C5a receptor 1. J. Immunol..

[10] Dave B (2014). Targeting RPL39 and MLF2 reduces tumor initiation and metastasis in breast cancer by inhibiting nitric oxide synthase signaling. Proc. Natl Acad. Sci. USA.

[11] Fancello L, Kampen KR, Hofman IJF, Verbeeck J, Keersmaecker KD (2017). The ribosomal protein gene RPL5 is a haploinsufficient tumor suppressor in multiple cancer types. Oncotarget.

[12] Hong M, Kim H, Kim I (2014). Ribosomal protein L19 overexpression activates the unfolded protein response and sensitizes MCF7 breast cancer cells to endoplasmic reticulum stress-induced cell death. Biochem. Biophys. Res. Commun..

[13] He H, Sun Y (2007). Ribosomal protein S27L is a direct p53 target that regulates apoptosis. Oncogene.

[14] Li J (2007). Ribosomal protein S27-like, a p53-inducible modulator of cell fate in response to genotoxic stress. Cancer Res..

[15] Xiong X (2014). Ribosomal protein S27-like is a physiological regulator of p53 that suppresses genomic instability and tumorigenesis. eLife.

[16] Zhao Y, Tan M, Liu X, Xiong X, Sun Y (2018). Inactivation of ribosomal protein S27-like confers radiosensitivity via the Mdm2-p53 and Mdm2–MRN–ATM axes. Cell Death Dis..

[17] Peterson TR (2009). DEPTOR is an mTOR inhibitor whose frequent overexpression in multiple myeloma cells promotes their survival. Cell.

[18] Zhao Y, Xiong X, Sun Y (2011). DEPTOR, an mTOR inhibitor, is a physiological substrate of SCF(βTrCP) E3 ubiquitin ligase and regulates survival and autophagy. Mol. Cell.

[19] Gao D (2011). mTOR drives its own activation via SCF(β-TRCP)-dependent degradation of the mTOR inhibitor DEPTOR. Mol. Cell.

[20] Duan S (2011). mTOR generates an auto-amplification loop by triggering the βTrCP- and CK1α-dependent degradation of DEPTOR. Mol. Cell.

[21] Mizushima N (2007). Autophagy: process and function. Genes Dev..

[22] Yorimitsu T, Klionsky DJ (2005). Autophagy: molecular machinery for self-eating. Cell Death Differ..

[23] Debnath J (2011). The multifaceted roles of autophagy in tumors—implications for breast cancer. J. Mammary Gland Biol. Neoplasia.

[24] Jain K, Paranandi KS, Sridharan S, Basu A (2013). Autophagy in breast cancer and its implications for therapy. Am. J. Cancer Res..

[25] Mizushima N, Yoshimorim T, Levine B (2010). Methods in mammalian autophagy research. Cell.

[26] Kim YC, Guan KL (2015). mTOR: a pharmacologic target for autophagy regulation. J. Clin. Invest..

[27] Burnett PE, Barrow RK, Cohen NA, Snyder SH, Sabatini DM (1998). RAFT1 phosphorylation of the translational regulators p70 S6 kinase and 4E-BP1. Proc. Natl Acad. Sci. USA.

[28] Sarbassov DD, Guertin DA, Ali SM, Sabatini DM (2005). Phosphorylation and regulation of Akt/PKB by the rictor-mTOR complex. Science.

[29] Li B, Dewey CN (2011). RSEM: accurate transcript quantification from RNA-Seq data with or without a reference genome. BMC Bioinformatics.

[30] Artero-Castro A (2015). Disruption of the ribosomal P complex leads to stress-induced autophagy. Autophagy.

[31] Heijnen HF (2014). Ribosomal protein mutations induce autophagy through S6 kinase inhibition of the insulin pathway. PLoS Genet..

[32] Shukla S (2014). KRAS protein stability is regulated through SMURF2: UBCH5 complex-mediated β-TrCP1 degradation. Neoplasia.

[33] Wei S (2012). Targeting the oncogenic E3 ligase Skp2 in prostate and breast cancer cells with a novel energy restriction-mimetic agent. PLoS ONE.

[34] Kuang P, Tan M, Zhou W, Zhang Q, Sun Y (2016). SAG/RBX2 E3 ligase complexes with UBCH10 and UBE2S E2s to ubiquitylate β-TrCP1 via K11-linkage for degradation. Sci. Rep..

[35] Qu X (2003). Promotion of tumorigenesis by heterozygous disruption of the beclin 1 autophagy gene. J. Clin. Invest..

[36] Finak G (2008). Stromal gene expression predicts clinical outcome in breast cancer. Nat. Med..

[37] Wang S (2010). RPL41, a small ribosomal peptide deregulated in tumors, is essential for mitosis and centrosome integrity. Neoplasia.

[38] Reza AMMT (2017). MicroRNA-7641 is a regulator of ribosomal proteins and a promising targeting factor to improve the efficacy of cancer therapy. Sci. Rep..

[39] Huang CJ (2013). Ribosomal protein S27-like in colorectal cancer: a candidate for predicting prognoses. PLoS ONE.

[40] Zhao Y, Xiong X, Jia L, Sun Y (2012). Targeting Cullin-RING ligases by MLN4924 induces autophagy via modulating the HIF1-REDD1-TSC1-mTORC1-DEPTOR axis. Cell Death Dis..

[41] Su H (2017). VPS34 acetylation controls its lipid kinase activity and the initiation of canonical and non-canonical autophagy. Mol. Cell.

