# Supplementary material for: Ribosomal protein S27-like regulates autophagy via the β-TrCP-DEPTOR-mTORC1 axis
Source: Cell Death Dis. 2018 Nov 13;9(11):1131. doi: 10.1038/s41419-018-1168-7 (PMC6234217; doi:10.1038/s41419-018-1168-7)

Supplemental Materials

Ribosomal protein S27-like regulates autophagy via the β-TrCP-DEPTOR-mTORC1 axis

Xiufang Xiong[1*#], Xia Liu[1#], Haomin Li[1], Hengqian, He[1], Yi Sun[1,3*], and Yongchao Zhao[1,2*]

FIGURE LEGENDS

Figure S1. Silencing of RPS27L induces autophagy in breast cancer cells.
(A) Autophagy measured by LC3-II conversion and p62 degradation. Cells were transfected with scramble control siRNA (siCont) or another siRNA oligos targeting RPS27L (siRPS27L-2) for 48 h, followed by IB with indicated Abs. SE: shorter exposure; LE: longer exposure. (B) Autophagy induction by RPS27L silencing was blocked by BAF A1 treatment. Cells were transfected with indicated siRNA oligos for 48 h, and then left untreated or treated with 50 nM BAF A1 for 24 h, followed by IB with indicated Abs. (C) No autophagy induction by RPS27 silencing. Cells were transfected with scramble control siRNA (siCont) or another siRNA oligos targeting RPS27L or RPS27 for 48 h, followed by IB with indicated Abs. (D) Reduced cell growth upon RPS27L silencing. Cells were transfected with scramble control siRNA (siCont) or another siRNA oligos targeting RPS27L for 48 h, followed by IB with indicated Abs (top panel) and ATPlite assay (bottom panel). Data shown are mean ± SEM. n=3, $^*p<0.05$, $^{**}p<0.01$.

Figure S2. Silencing of RPS27L inhibits the phosphorylation of S6K1 triggered by TPA.
Cells were transfected with indicated siRNA oligos for 48 h, and then serum starved for 24h, followed by treatment with 10ng/ml TPA for indicated time periods. Cells were then harvested for IB with indicated Abs.

Figure S3. *Rps27l* disruption inhibits mTORC1 activity in MEF cells.
Another independent pair of MEFs cells was generated from different pregnant mouse, followed by IB with indicated Abs.

Figure S4. Blockage of autophagy by BAF A1 treatment significantly induces cell apoptosis upon silencing of RPS27L.
Cells were transfected indicated siRNA oligos for 48 h, and then left untreated or treated with 50 nM BAF A1 for 24h, followed by IB with indicated Abs.

Xiong et al. Figure S1

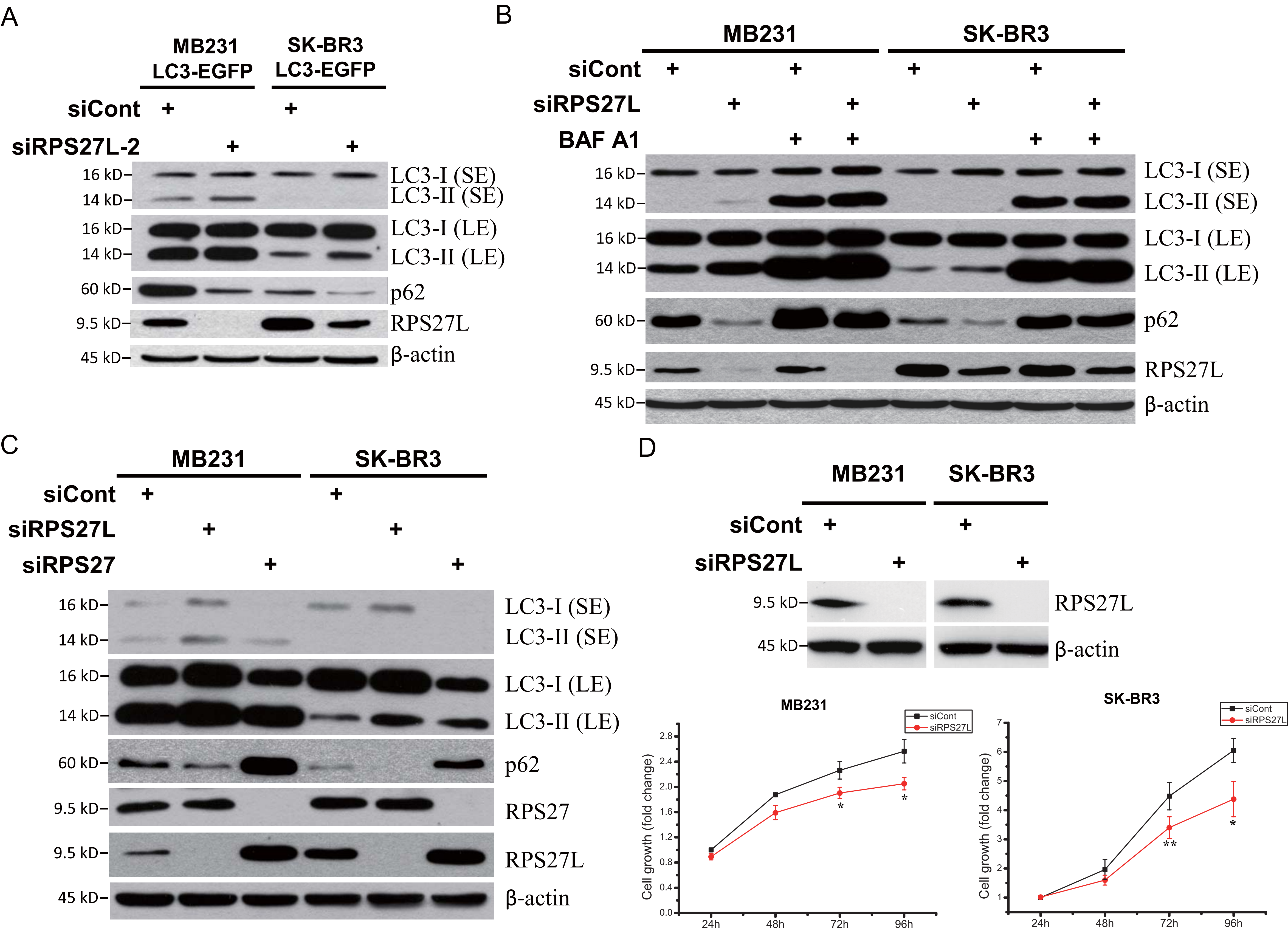

Xiong et al. Figure S2

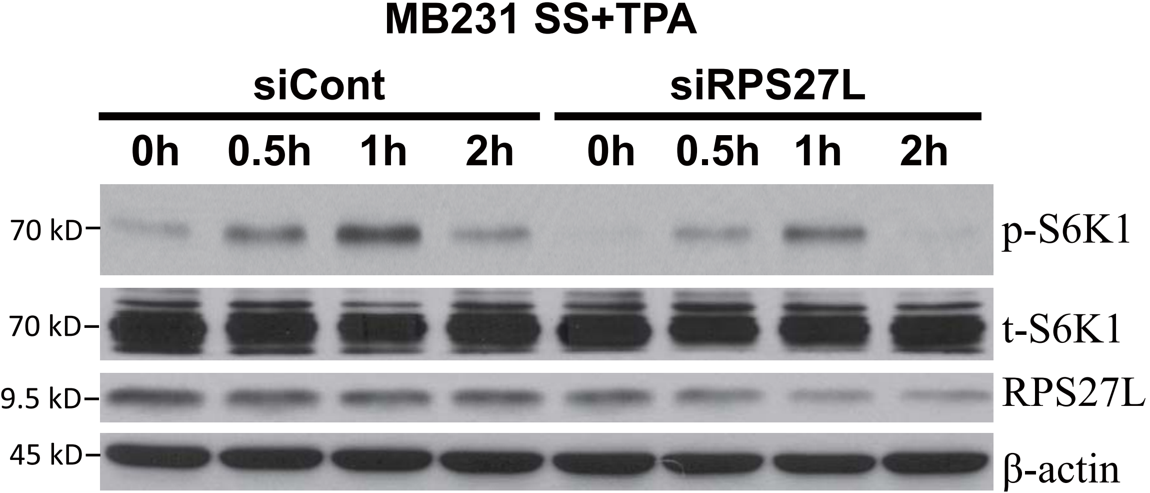

Xiong et al. Figure S3

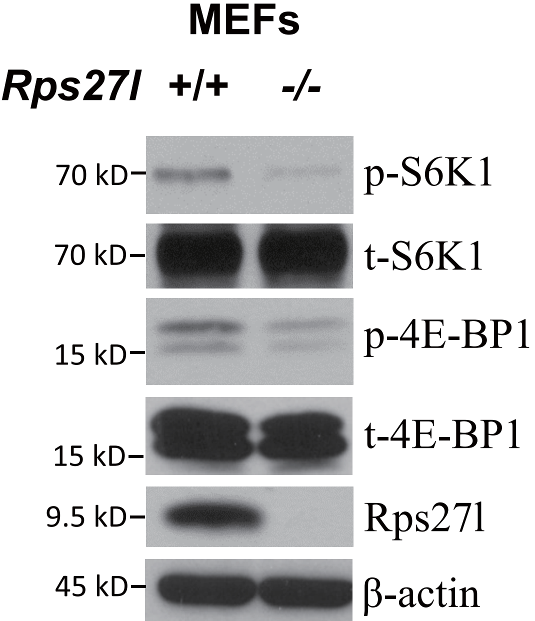

Xiong et al. Figure S4

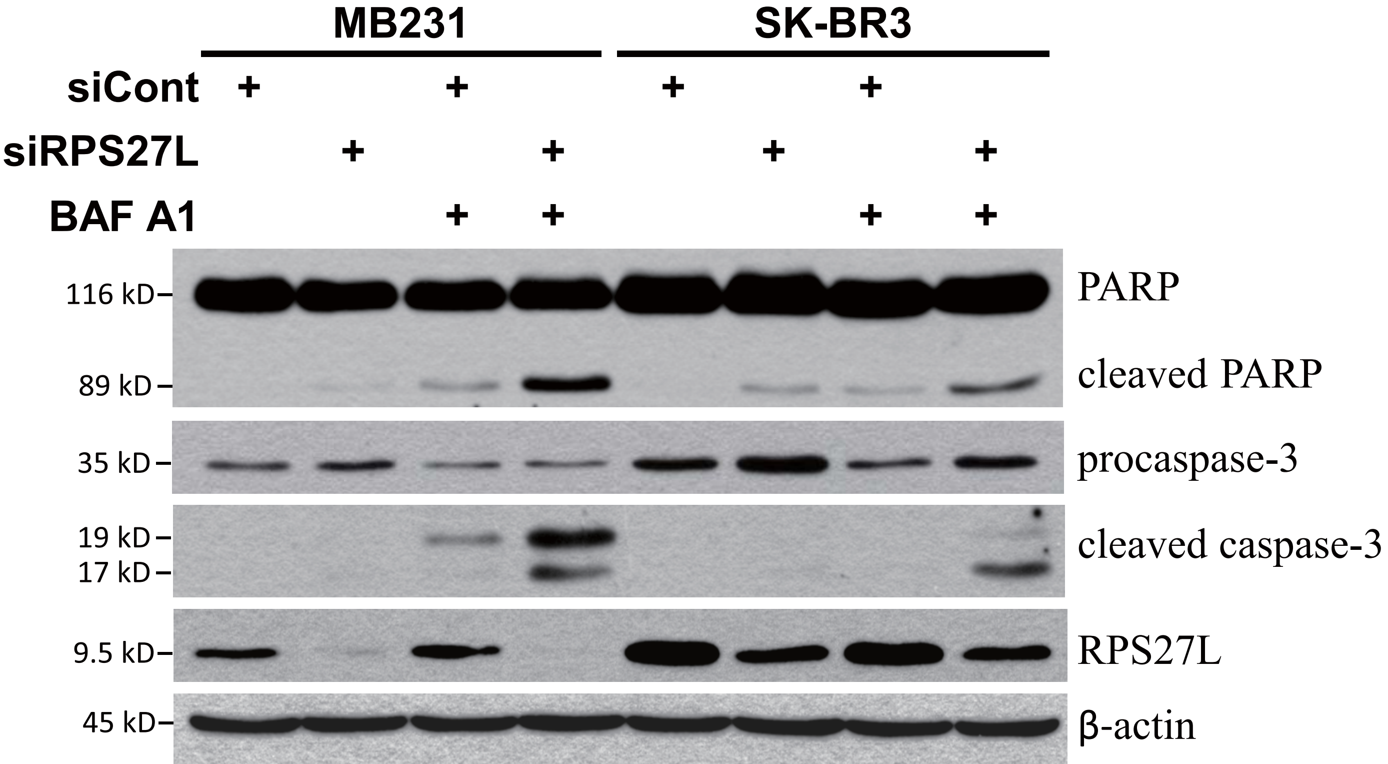

Supplement: Supplementary file 1 — Supplemental material [file 41419_2018_1168_MOESM1_ESM.pdf]
